# Strengthening the community health program in Liberia: Lessons learned from a health system approach to inform program design and better prepare for future shocks

**DOI:** 10.7189/jogh.11.07002

**Published:** 2021-03-10

**Authors:** Aline Simen-Kapeu, Sonia Lewycka, Ochiawunma Ibe, Anthony Yeakpalah, Jannie M Horace, Geneviève Ehounou, Tamba Boima, Chea Sanford Wesseh

**Affiliations:** 1 United Nations Children’s Fund, Regional Office, Senegal; 2 Centre for Tropical Medicine and Global Health, University of Oxford, UK; 3 ICF Rockville, Maryland, USA; 4 United Nations Children’s Fund, Monrovia, Liberia; 5 United States Agency for International Development, Monrovia, Liberia; 6 Ministry of Health, Monrovia, Liberia

## Abstract

**Background:**

Arising from the Ebola virus disease (EVD) outbreak, the 2015-2021 Investment Plan aimed to improve the health status of the Liberian population through building a resilient health system that contributes to achieving equitable health outcomes. Recognizing the significance of community participation in overcoming the EVD outbreak, strengthening community systems emerged as one of the most important strategies for bridging the gap in accessing primary health care (PHC) services. This study reviewed the community health policy development process in order to draw lessons from the health system strengthening efforts in Liberia post-EVD crisis.

**Methods:**

A government-led health system analysis approach was applied to assess, review and revise the community health program in Liberia. The mixed method approach combines the use of an adapted tool to assess bottlenecks and solutions during workshops, a qualitative survey (key informant interviews and focus group discussions) to assess perceptions of challenges and perspectives from different stakeholders, and an inter-agency framework – a benchmarks matrix – to jointly review program implementation gaps using the evidence compiled, and identify priorities to scale up of the community program.

**Results:**

Stakeholders identified key health system challenges and proposed policy and programmatic shifts to institutionalize a standardized community health program with fit for purpose and incentivized community health assistants to provide PHC services to the targeted populations. The community health program in Liberia is currently at the phase of implementation and requires strengthened leadership, local capacities, and resources for sustainability. Lessons learned from this review included the importance of: establishing a coordination mechanism and leveraging partnership support; using a systems approach to better inform policy shifts; strengthening community engagement; and conducting evidence-based planning to inform policy-makers.

**Conclusions:**

This article contributes toward the existing body of knowledge about policy development processes and reforms on community health in Liberia, and most likely other African settings with weak health systems. Community-based systems will play an even bigger role as we move toward building resilience for future shocks and strengthening PHC, which will require that communities be viewed as actors in the health system rather than just clients of health services.

In 2014-2015, Liberia experienced the most devastating Ebola virus disease (EVD) outbreak in history, which was preceded by a prolonged civil conflict. These two tragic periods impacted Liberia’s fragile health system, resulting in the near collapse of the health workforce, through the flight and/or death of health-workers. The EVD outbreak called for a re-examination of Liberia’s national health system, health policies, and plans [1,2].

Systems thinking has emerged as an important approach to health programming, that reveals relationships and synergies affecting the delivery of priority health services [3]. A holistic understanding of a health system’s building blocks [3] better informed the identification of challenges to strengthen the health system. Definitions of health systems strengthening have been limited in their inclusion of communities, despite evidence that community involvement improves program effectiveness for many health interventions. Community ownership and partnerships were included in the WHO’s health systems' building blocks following recommendations of the Ouagadougou declaration on primary health care in Africa [3,4], which reaffirmed the importance of involving and empowering communities in health development.

Community engagement in health systems is both a practical response to the challenging conditions of health provision in low-income settings and a key principle for strengthening health systems more generally [5,6]. The need for integration of communities in planning, delivering, and evaluating health services has become even more apparent with recent infectious disease outbreaks, including Ebola and COVID-19 [1,2,6]. Today systems are called upon to not only respond to unanticipated shocks, such as epidemics or humanitarian emergencies, but also to meet the challenge of achieving the new sustainable development goals (SDGs) for health [7,8]. Evidence suggests effective community health programs must be designed from a health systems perspective to be successful [6,9-11].

Implementing community health programs demands strong leadership and cross-sectoral coordination at all levels; human resources availability and capacity; sufficient funding; functional quality assurance and improvement mechanisms; reliable information management systems; and uninterrupted drug supply, particularly for populations living in remote areas [10,11]. Challenges associated with quality supervision and insufficient community sensitization and dialogue have undermined the impact of some community health programs [12,13]. Community health programs may also face a challenging environment where community health workers are seen as a second-rate option for service delivery, or where mechanisms for their motivation may cause controversy [10,13].

## Overview of the community health program development process in Liberia

**Figure 1:**
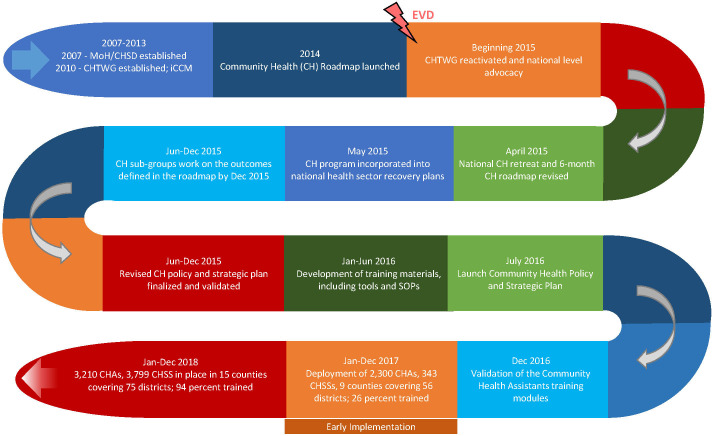
The community health development process in Liberia (2007-2018), which Includes the review process post EVD (2015-2016).

The community health program development process in Liberia is presented in **Figure 1**. The process started in 2007 (pre-Ebola), with the establishment of coordination and technical committees. During this period, the Community Health Roadmap was developed, and the Integrated Community Case Management (iCCM) program was piloted, to assess the feasibility and effectiveness of implementing a community health program. The iCCM program is a strategy to increase access to effective case management of malaria, pneumonia and diarrhea among children under five years, especially in remote and hard to reach areas. The pilot iCCM program (2010-2011) showed that general community health volunteers (gCHVs) could deliver the services defined in the policy, with adequate supervision and supplies [14].

However, despite the ability to reach scale across rural communities, the iCCM program faced challenges in quality and management [15]. Effective implementation was limited through major bottlenecks, such as frequent stock-out of medicines, poor data management, ineffective supervision, lack of motivation/incentives, limited community involvement, and fragmented programming [6]. In 2011, the Community Health Policy was revised to reinforce implementation of the essential package of health services (EPHS) [16,17] and strengthen existing community health structures [16]. This revision was informed by analysis of key program documents and reports from government and partners, highlighting lessons learned from the field.

The EVD outbreak of 2014-2015 weakened Liberia’s health system further, which was ill equipped to effectively respond to the epidemic. Primary health care (PHC) services were rendered dysfunctional, with health facility closures caused by fear of being infected, health-worker absenteeism, and community distrust and fear [18]. At the onset of the outbreak, the health system mounted a response that had limited community engagement and participation, which likely reduced the effectiveness of the EVD response and accelerated the spread of EVD cases in the community [19].

In 2016, based on lessons learned from the EVD outbreak period, and previous efforts to implement the community health program in the country, the Government of Liberia and its development partners, developed a revised national policy on Community Health Services. Two key priorities were to 1) develop an incentivised community health workforce to increase community-based service delivery in remote areas, and 2) ensure an enabling environment that restores trust in the health authorities’ ability to provide services through community engagement [20].

The aim of this article is to summarize this community health policy development process and review progress made and lessons learned in strengthening the community health system in Liberia post-EVD crisis, between January 2015 and July 2016.

## METHODS

### Community health development process in Liberia

In January 2015, the community health technical working group (CHTWG) was reactivated and several sub-groups (Recruitment and Remuneration; Training & Supervision; Supply Chain, and Monitoring & Evaluation), headed by various Ministry of Health (MoH) departments, were tasked with revising the Community Health Policy in line with the *Investment Plan for Building Resilient Health Systems* in Liberia (2015-2021). A systematic methodology was adopted.

The health systems approach was applied to revise the existing community health policy, and to re-establish the community health system with an appropriate, well-trained, supervised, and incentivized cadre of community workers to provide PHC services to populations with limited health care access. The community health program in Liberia was developed through a government-led process (**Figure 1**), and used a combination of sources of evidence, including bottleneck analysis, a qualitative study, and benchmarks matrix [21].

### Health system bottleneck analysis

Country stakeholders met during a series of program review meetings held in March/April 2015 in all 15 counties of Liberia. The workshops followed a pre-defined agenda, where stakeholders assessed the bottlenecks to scaling up community health programs and discussed solutions to address them. An adapted Tanahashi framework was used to assess health system bottlenecks linked to the following determinants: availability of commodities, availability of human resources, geographic accessibility, utilization (initial and continuous) of services, demand for services (community engagement), and quality of care [22,23]. This approach has previously been used to identify bottlenecks in reproductive, maternal, newborn and child health (RMNCH) service delivery [23]. Assessment of the enabling environment, including the functionality of the community health structures, was done, to understand the potential impact on service delivery. Workshop participants used available program reports from MoH, administrative data, and professional experiences to identify the health system bottlenecks to scale up community health. Then participants proposed potential strategies to address identified bottlenecks. The CHTWG reviewed workshop reports and identified common issues raised by participants for each domain of the health system. During a 3-day retreat in Bong county in April 2015, MoH program managers and designated CHTWG sub-group members collated and shared the first analyses with all participants for discussion; this further informed the revision of the Community Health Policy which was later revised and validated in December 2015 [24].

### Qualitative study

Between September and November 2015, a qualitative study was conducted in five counties (Bomi, Bong, Grand Gedeh, Montserrado, and River Cess), to explore stakeholders’ opinions and perceptions on strengthening the community health program. Methods included focus group discussions (FGDs) and in-depth interviews (IDIs). A two-day training was conducted for interviewers, field coordinators, and note-takers, and covered field operations, ethics, interviewing techniques, transcription, and safety. The semi-structured interview guide was developed through review of previous research interviews and consultations with experts involved in community health programs. It included the thematic areas of policy and strategy development, coordination, performance management, and facilitators and barriers for the planning and/or implementation of the community health strategy. One county was randomly selected from each of the five health regions of Liberia. Participants for IDIs were selected through purposive sampling, and included policy-makers, program managers, health care workers involved in community health programs, and County/District Health Team members. Participants for FGDs included gCHVs, community leaders/representatives, pregnant women, and mothers and fathers of children under 5 years.

Five teams, each comprising one interviewer and two note-takers, conducted the data collection through interviews, of 90 minutes each, over a two-week period. Interviews were audio-recorded, transcribed, and entered into NVivo (version 9). Analyses were carried out using a general inductive approach to systematically summarize views regarding specific research questions, rather than seeking to develop a new theory, or describe a phenomenon or lived experience [25]. Data were read with the research areas in mind, but no *a priori* models were imposed. Contents were aggregated across interviews, and lower order units of meaning were identified and clustered into themes and sub-themes. Data were coded using these themes, and quotes encapsulating themes were selected. Ethical approval for this study was obtained from the University of Liberia Institutional Review Board. Verbal consent was sought from participants, after explaining the study and assuring confidentiality.

### Benchmarks matrix framework

The iCCM interagency framework has been used to assess progress made in planning and implementing community health programs [26]. Based on the learning from the EVD outbreak that had a negative impact on health services, the MoH in Liberia expanded the package of care for children from iCCM to a broader package of RMNCH services including community-based surveillance. The benchmarks matrix proposed by McGorman et al uses a health systems approach, and offers insights on the design and implementation level of community programs [26]. The interagency framework includes eight health systems components: coordination and policy setting; costing and financing; human resources; supply chain management; service delivery and referral; communication and social mobilization; supervision and performance quality assurance; and monitoring and evaluation. These components mirror the six WHO health systems' building blocks with the addition of communication/social mobilization and supervision/quality assurance. The benchmarks are grouped into three phases: advocacy/planning, pilot/early implementation, and scale-up [26].

The CHTWG met several times in 2015 to review documents shared with the government by partners (program/research reports, published and unpublished articles, presentations, and policy documents), highlighting bottlenecks and achievements of the community health program during the past 10 years. The CHTWG compared these findings with results from the bottleneck analysis and the IDIs/FGDs, using the domains of the benchmarks matrix, to identify common issues, review achievements against the benchmarks, and assess the community health program implementation.

## RESULTS

### Stakeholder-led health system bottleneck analysis

There were more than 200 workshop participants in total (10-20 people per county; aged 21-55 years; around 30% women). They were members of the regular health technical committees consisting of MoH and Ministry of Planning representatives, experts from United Nations agencies, professional bodies, non-governmental organizations (NGOs), health facility staff, and local authorities. They were experts from diverse fields nominated by the government to provide ongoing advice on community health issues. **Table 1** summarizes key health system bottlenecks identified to the scale up of the community health interventions.

**Table 1:** Key bottlenecks to scaling up the community health program identified by stakeholders in Liberia, 2015

Health system areas	Key bottlenecks to scale up community health
**Enabling environment:**	
Poor governance	Inadequate oversight of Ministry of Health (MoH) – poor coordination mechanisms leading to partners implementing ad hoc and vertical community programs
Weak management	Weak management capacities at the county and district levels, especially poor planning and monitoring mechanisms
Inadequate policy and legal framework	Policy fell short on community-based surveillance of events, and standard strategies on community engagement; Weak enforcement of the policy due to lack of an operational plan
Limited funding	No government budget line to support implementation of community health interventions
Poor accountability	Poor involvement of community networks in Reproductive, Maternal, Newborn, Child and Adolescent Health-related activities due to the weak functioning of community structures
**Availability of supplies and commodities:**	
Weak coordination and leadership	Weak coordination at the district and facility levels: Parallel distribution of community supplies; Strong influence of partners implementing vertical programs; poor engagement with pharmacists and the central unit
Inefficient distribution	Inconsistency in availability of drugs and commodities in remote areas due to frequent stock-outs as a result of poor supply chain management at county, district, health facility, and community levels
Inadequate inventory management	Product flow: 1) *lack of inventory management*: inefficient distribution of drugs due to frequent delays in getting requisition and consumption products from county level; 2) *storage practices and conditions*: gCHVs used inappropriate storage practices
	Data flow: 1) *Availability*: Consumption data are non-existent or inaccessible by any level of the supply chain; Visibility into stock levels at gCHV level by decision makers throughout the system is limited or non-existent; 2) *Accuracy*: facility consumption data are inaccurate, does not consider the community level in most counties and can’t be used for resupply planning
**Availability of community health workforce (human resources):**	
Lack of job description	Diversity of community cadres providing separate services which are poorly coordinated; Lack of clear job description and recruitment guidelines for gCHVs
Lack of coordination on training	Non-uniform training packages supported by different MoH departments for community cadres (mainly gCHVs and Trained Traditional Midwives) In-service training for gCHVs done on an ad hoc basis based on donor funding/priorities; Lack of coordination by Government and partners on duration and content
Inconsistent incentives	Non-monetary incentive policy in place but not consistently followed by partners; Uneven motivation package for CHVs implemented by partners leading to high attrition rate
**Delivery of community health services (accessibility):**	
Poor geographic accessibility	Due to lack of availability of key maternal, newborn, and child health services in remote communities; Weak linkages between communities and the existent health facilities
Inadequate service integration	Frequent interruption of outreach services in far to reach areas due to limited funding and inadequate planning
**Utilization of services:**	
Low awareness	Low awareness at the community level; cultural barriers
Transport barriers	Transport/ distance barriers to health facilities
**Demand for services / Community engagement:**	
Weak community structures	Many local leaders have not been involved in community health activities; most community health boards are not functional
Coordination and linkages	Lack of coordination between community structures, interventions and services with many vertical efforts ongoing; Poor community linkages with the health service delivery system
Trust	Loss of community trust in health system due to the EVD outbreak
**Quality of services, including monitoring and evaluation and surveillance systems:**	
Weak supervision	Lack of supervision systems in place for gCHVs; Limited number of Community health focal points positions mainly at the district level
Poor quality assurance systems	Lack of operating manuals, standard operating procedures, job aids/job description for different community cadres
Inadequate monitoring and evaluation	No national monitoring and evaluation framework to monitor progress and measure impact of community health interventions; Monthly data submitted by a limited number of gCHVs and limited reporting by MoH, mostly through non-governmental organizations; There is no structured system for community-based disease surveillance. Initial reporting and recording tools were developed, but had not been implemented, disseminated or utilized; community ledgers were not uniformly distributed, and health facility reports do not include the community level; Maternal and neonatal deaths not captured in community-based reports

For the ***enabling environment***, the main challenges identified included the community health policy gaps on community-based surveillance and community engagement; the lack of a national budget line and clearly earmarked domestic resources for community health; and the lack of a budgeted operational plan to support implementation. Most community-based initiatives were donor-driven and county-specific; Stakeholders also identified that weak governance and limited management capacities at the national, county and district levels, had led to implementation of donor-driven, vertical community interventions in selected counties, with very limited involvement of community structures and local government entities.

The ***supply chain management*** issues were linked to 1) weak coordination and leadership at the central unit, and poor links with pharmacists; 2) inefficient distribution of drugs in remote areas due to frequent delays in getting products from county level and inadequate storage practices and conditions; 3) inadequate inventory management with lack of community-level consumption data. For the ***human resource*** component, the diversity of profiles for community cadres, and the non-uniformity of the training packages, including in-service training, were major challenges. Most trainings were organized based on donor funding and priorities, making it difficult to make a coordinated assessment of the performance and contribution of community health cadres to the health system. In relation to ***service delivery,*** community-based maternal, newborn, and child health services were limited in remote areas mainly due to lack of service integration, poor geographic accessibility to health facilities, and non-functional referral systems. ***Utilization*** was affected by low awareness, cultural barriers and transport. In terms of c***ommunity engagement,*** key bottlenecks were weaknesses in the functioning of existing community structures and poor involvement of community leaders and networks. Most community health committees were not functional and the lack of linkages between the communities and the health system was exacerbated by the loss of community trust in the health system due to the EVD outbreak.

The main challenges related to ***quality of care*** were the lack of quality assurance systems including operational manuals, standard operating procedures, job aids, and poor functioning supervision mechanisms to support community health cadres. The EVD outbreak revealed that there was no structured system for community-based disease surveillance. We found that although initial reporting and recording tools were developed for the iCCM program, the information system had not been adequately established. In addition, there was no standard M&E framework to monitor progress. Information was collected from a limited number of gCHVs mainly by vertical programs.

### Qualitative study on perceptions and needs for the community health program

A total of 4 IDIs and 5 FGDs were conducted per county (20 and 25 in total respectively). FGDs had 6-10 participants each. The qualitative survey provided information on community and stakeholders’ perceptions and needs regarding components of the community health program. Five over-arching themes emerged (**Table 2**).

**Table 2:** Themes and sub-themes emerging from the qualitative study with illustrative quotes

Theme 1: Leadership and governance:
1.1 Importance of community health policy:
“Ah it’s important because this is a strategy that will lead us to achieve those goals we set. I mean we, as health institution or our community health program…because the strategy will tell us what processes, how we are going to get to where we want to go.” [Program Manager, Bong]
1.2 Prioritization of community health post-Ebola virus disease (EVD):
“Before 2015, we recognized that access to basic health services was low, so the community health program was established to make sure that services reach out to these populations so that we can be able to reduce under five and maternal mortality. It became paramount when we recognized that the role played by communities and community members in the EVD was so significant that we thought it was important to think about community health programs.” [Program Manager, Montserrado].
**Theme 2: general community health volunteer (gCHV) motivation:**
2.1 Lack of standardized incentives:
‘’One of the greatest challenges is the lack of standardized incentives for the community volunteers.” [IDI, Montserrado]
‘’I am not holding it against anybody if it is God’s will. During my training, I was told, I was not going to be paid. But even so, as a human, considering the distances that we are covering, we should be given at least Motorcycle or Bicycle to get there. Since we have been working here from that time, now Government should be doing something about us. We are not demanding money, but give us motorcycle to get to where we are supposed to go and work, that’s all. But I really love the job.” [gCHV, Bomi]
2.2 Pride:
‘’The letter was sent to my community with all information in it. And they called the Town meeting they explained the letter in detail, I felt proud, from that day I got passion for the job up to present.” [gCHV, Bomi].
2.3 Helping others:
‘’What encourages me about general CHV is that, it makes me popular in the community. I like that and more besides, it makes me feel that I am really helping my people. I am one single person helping more than thousands and they take me in high esteem.” [gCHV, Bong]
2.4 Personal knowledge:
“What I like about being a gCHV is that I learn more things because when I was not gCHV I don’t know the danger sign on a child even I have my own child, if the child is [convulsing], I feel that different thing happening to my child, but now I know the importance of health and I know the importance of going to the hospital, so I am proud.” [gCHV, Grand Gedeh]
**Theme 3: Policy:**
3.1 Previous policy gaps:
“Actually, the policy was done and there were key areas that were not included in the policy. There was no EVD, and the issue of infection prevention and control, surveillance and also community engagement were not included in the policy.” [Manager, Montserrado].
**Theme 4: Quality service delivery:**
4.1 Mis-match between gCHV training and delivery of services:
“We were trained to take malaria test, to take temperature to see you know how the child’s temperature is, whether he has malaria plus what or what...then refer them, but nothing like that. That is what we are supposed to be doing, but nothing. One of the task was to give family planning to those young girls, but I can say over one year now, it has been cut off, and you can find them in the communities now, there are many drop-outs now from schools now.” [gCHV, Montserrado]
“Even to identify a malnourished child. Those are things that we are supposed to be doing but we are not doing them.” [gCHV, Bong]
4.2 Competing priorities of gCHVs:
“For me when my child is sick, I can rush to the clinic because, the reason there is gCHV every day will not be by me because, she’s not receiving pay. Sometimes she will go to her daily activity that will be able to give her lay cash to sustain herself. So in sitting to wait (claps) you might be at risk. So when the child is sick, sometime you may not see her and you can’t sit and wait for her say let she come anyhow and give me advice, so I can just rush to the clinic”. [Mother, River Cess]
4.3 Referrals
“When your child is sick we can go to see the gCHV. He will advise you what to do for the child. Then when the sickness still continues then he is the one who will transfer you to the hospital” [Mother, Grand Gedeh]
**Theme 5: Community engagement:**
5.1 Importance of the community post-EVD:
“Ebola taught us many lessons and we realized that the community was an important component of the health system that helps reduce the burden or decrease the Ebola burden in the country”. [Manager, Bong].
5.2 Need for community health policies post-EVD:
‘’What role can they play? How can they get involved into community health programs, you saw what happened during the EVD, most of them organized themselves into task forces and responded, so these structures should be actually policy-based”. [Manager, Montserrado]

***Leadership and governance.*** Key informants recognized the importance of the community health policy as a foundation to strengthen implementation of health programs. They also described how the EVD outbreak was the dominant factor that influenced the prioritization of community health issues. The role played by communities (community-based surveillance, information and education campaigns, health and hygiene promotion, contact tracing, alert and referral of cases, support to safe burials) across the country during this Ebola period was very significant, and this was a driving force in the decision to review and revise the 2011 community health policy.

***gCHV training and motivation.*** There were varying experiences among gCHVs in terms of the duration of their initial training to become community volunteers. The lack of standardized trainings, incentives, and supplies were reported as major challenges to be addressed in the community health policy. Despite challenges, the primary motivating force to serve as gCHVs, was a sense of pride at being selected by their community for the position. They emphasized on their commitments to serving their community. Several participants also mentioned the personal knowledge gained as a benefit of being a gCHV.

***Policy.*** Key Informants at the MoH averred that community-based surveillance and infection prevention and control (IPC) were major gaps in the 2011 policy.

***Quality service delivery and supply chain management.*** gCHVs felt there were tasks they should be doing but were not doing, centered primarily around dispensing medicine to treat sick children or providing basic family planning services. However, the respondents acknowledged the gCHV’s role in promoting general medicine over traditional medicine. Pregnant women and mothers felt that gCHVs are not well equipped to carry out their preferred functionalities. They recognized their presence in their communities but had mixed views on immediate care-seeking due to the flexibility of the gCHV’s agenda.

***Community engagement.*** Key respondents noted that community engagement, ownership, and empowerment of gCHVs were not considered during program implementation and were major gaps in the 2011 community health policy. Acknowledging the volunteer nature of the gCHVs’ job, respondents suggested that communities sharing food with gCHVs would show appreciation and reciprocity for their work. Respondents also suggested pooling community funds to offset the purchase of medicine that gCHVs sometimes supply, and to help transport sick community members to the hospital.

### Community health policy shifts approved by policy-makers and stakeholders

In 2015 the CHTWG was functional and regularly guiding and supporting the Community Health Services Division. All strategies and recommendations proposed by stakeholders to address identified bottlenecks, were reviewed in multiple occasions, including at the 3-day retreat and later. Consensus was achieved at the end and informed the development of key programmatic shifts described in **Table 3** which guided the revision of the 2016 community health policy. Major changes were made in the following domains: 1) Human resource: a new cadre ‘Community Health Assistants (CHAs)’ to operate in remote communities to provide access to basic RMNCH services; 2) new standardized community health service delivery package; 3) a standardized incentive and motivation scheme; 4) a standardized training modalities and curriculum based on the new package of services; 5) a community supply chain system integrated into the existing national supply chain; 6) a community-based information system to be integrated into the national district health information system (DHIS2); 7) the development of a community event-based surveillance system as part of Integrated Diseases Surveillance and Response (IDSR) activities; 8) Quality assurance systems defined, including a newly defined cadre of CHA supervisors named Community Health Services Supervisor (CHSS). The draft of the policy was further submitted for clearance to the MoH high-level managers.

**Table 3:** Key community health policy shifts, 2016

Health systems areas	Community health policy shifts
Community health cadre	A new cadre ‘community health assistants (CHAa)’ to operate in communities located beyond 5 km from health facilities to provide access to basic Reproductive, Maternal, Newborn, Child and Adolescent Health services. CHAs will serve up to 40-60 households.
Service delivery package	New standardized community health service delivery package described in the new policy developed
Motivational incentives	Standardized incentive and motivation scheme
	• CHAs to receive US$70/mo as incentives
	• Community health volunteers (CHVs) could occasionally receive campaign DSA and non-monetary incentives
	• Performance based incentives could be piloted
Training program	Standardized, evidence-based training approach
	• Standardized pre-service curricula for CHAs with contents based upon service delivery package and scope of work for each cadre
	• Facilitator’s guide, learning and job aids, and supervision tools to be used during the training
	• In-service training/refresher training to be organized based on performance
Supply chain	Standardized, integrated supply chain
	• Standardized list of essential equipment for CHAs
	• Integration of the community supply chain into the existing national supply chain
	• Clearly defined distribution and delivery channels to CHAs
	• Medicines, medical supplies and other logistic needs for CHAs adequately quantified to prevent stock-outs
Monitoring and evaluation	Integrated community-based Information system
	• Functional community-based information system integrated into district system (DHIS2)
	• Data collection, tracking, and monitoring tools and mechanisms revised / developed
	• Develop monitoring and evaluation framework with clear indicators
	• Document best practices and lessons learned
Community-based surveillance	• Set-up community-event based surveillance system as part of Integrated Diseases Surveillance and Response (IDSR) activities
Quality of care including supervision	• CHAs to be supervised by a Community Health Services Supervisor (CHSS)
	• CHSS should be a professionally trained health worker assigned to the health facility and supervised by the Officer-in-Charge
	• CHSS shall provide field-based supervision to CHAs
	• One CHSS shall supervise up to 10 CHAs. For catchment areas with more than 10 CHAs operating, additional CHSS shall be recruited
	• Quality assurance and improvement mechanisms should be established to strengthen linkages between heath facilities and communities

### Benchmarks matrix framework for program review

The stakeholder analyses guided by the benchmarks matrix completed in 2016 (health facility self-assessments, community health self-assessments, and review of administrative data), revealed that Liberia is still at an early implementation phase of its community health program (**Table 4**). The following key components of the CHA program have been progressively strengthened during the 4 years post-EVD: 1) coordination and policy setting; 2) costing and financing; 3) human resources; 4) supply chain management; 5) service delivery and referrals; 6) communication, engagement and social mobilization; 7) supervision and quality assurance; 8) information system. Key stakeholders noted the need to develop a sustainable and costed national community health scale-up plan and mobilize domestic and donor resources; to improve the quantification, logistics and monitoring systems for commodities at the community level; to develop a comprehensive communication and social mobilization plan to strengthen engagement with local governments and community networks and build resilience and sustainability. The review showed that two components – communication engagement and supervision/quality assurance – are still at phase 1, the planning phase, and required substantive joint efforts at the strategic and operational levels.

**Table 4:** An overview of the community health program implementation status in Liberia (2016)

Components	Implementation of the Community Health Program		Implementation status
**Main activities achieved**	**Main activities pending**
Coordination and policy setting	• Ministry of health (MoH) leadership established community health services department engaging other relevant departments and line Ministries;	• Validation and endorsement of the general community health volunteers (gCHVs) strategic plan;	Phase 2: Early implementation
	• National coordination mechanism in place – Community health technical working group established, and regular meetings held;		
	• Mapping of partners, stakeholders and donors supporting the community health program completed;		
	• National community health policy revised: integrated community case management (iCCM) program expanded to a broad package of core plus additional services;		
	• Community health strategic plan and operational plan developed;		
	• Community based assessments and situation analysis conducted;	• National community health scale-up and sustainability plan to be developed;	
	• Policy dissemination plan developed and disseminated at all levels	• MoH leadership to be strengthened at all levels for sustainability	
Costing and financing	• The costing of activities for the community health program estimated by different partners supporting implementation of the program in selected counties;	• National costed plan for scaling-up the community health assistant (CHA) program and resource mobilization strategy to be developed	Phase 2: Early implementation
	• Financing gap analysis completed by partners for selected counties where the community health program will be implemented; national community health investment case completed;	• Earmarked MoH funding for community health still pending (no specific budget line);	
	• Finances for community health medicines and supplies secured; additional support from partners for limited periods;	• Long term strategy for sustainability and financial viability not yet developed;	
Human resources	• Estimated numbers, roles and expectations of CHAs and community health service supervisors (CHSSs), communities and referral service providers defined; Criteria for CHAs and CHSSs recruitment defined; community engagement to ensure communities are fully aware and play a clear role in supporting CHAs in their roles;	• CHA career ladder is not defined yet;	Phase 2: Early implementation
	• Training curriculum and plan for comprehensive CHA training developed; Assessment of national training institutions completed;		
	• CHA incentive/motivation plan in place; Training of CHAs with community and facility participation completed in selected counties.	• CHA training to be completed for the remaining areas in selected counties	
Supply chain management	• Appropriate community health medicines and supplies consistent with national policies and included in the essential drug list;	• Functionality of monitoring systems for stocks and medicines at the community level to be strengthened (there is substantial stock out periods for iCCM drugs)	Phase 2: Early implementation
	• Quantification and procurement plan for community health medicines and supplies completed and consistent with national policies;		
	• Inventory control and resupply logistic system for community health integrated into national supply chain system including standard operating procedures;		
	• Monitoring systems for stocks and medicines at all levels defined	• Logistic systems and resupply procedures developed and implemented not at scale (mainly for iCCM drugs)	
Service delivery and referral	• Guidelines for clinical assessment, diagnosis, counseling, management, and referral developed; Referral and counter referral system developed between health facilities and communities but not yet fully functional;	• Implementation of referral and counter referral system to be strengthened: ensure information flow from referral facility back to CHA with returned referral slip; ensure strong involvement of community members to facilitate referrals	Phase 2: Early implementation
	• Assessment, diagnosis and treatment of sick children by CHAs in selected counties; Timely receipt and treatment by existing CHAs in selected counties but frequent stockouts of medicines.		
Communication, engagement and social mobilization	• Community structures in place but to be strengthened;	• Advocacy activities for the expanded package of services to create an enabling environment to be developed	Phase 1: Advocacy and planning
	• *Community mobilization*: 1) Communication strategies and education messages developed by various programs; 2) Training materials and job aids to strengthen communication and health promotion for various programs developed; 3) Dialogue between existing community health cadres and communities is ongoing through vertical programs but need to be structured; 4) Role and expectations of community health committees, community leaders, and members defined;	• Comprehensive communication and social mobilization plan to be developed in line with the new package of services (including a monitoring and evaluation plan)	
	• *Promotion of recommended key family practices*: counseling cards available for specific interventions;	• Needs to strengthen engagement with local governments for sustainability.	
Supervision and performance quality assurance	• National quarterly monitoring and supervision in place. Appropriate supervision system for the new CHA program including checklists and other tools developed;	• Quality assurance and performance mechanisms for the full package to be developed;	Phase 1: Advocacy and planning
	• Supervision plan, including number of visits, supportive supervision roles, self-supervision established; Supervisors trained in supervision and provided access to appropriate supervision tools in line with the new CHA program	• Supervision system to be strengthened	
Monitoring and evaluation; Heath information system	• Monitoring framework for all components developed and sources of information identified; Indicators and standards for community-based information system (CBIS) defined;	• Comprehensive monitoring and evaluation plan (to complete the monitoring framework) to be developed for the CHA program	Phase 2: Early implementation
	• Data source and tools for routine monitoring through the national health information system defined and established (linkage with district system - DHIS2);	• Development of national and sub-national score cards/ dashboards including community-based indicators	
	• Registers and reporting documents finalized with all data collection and tracking forms; Responsibilities by level of the health system and standard operating procedures available.	• Agenda on implementation research to be developed	
	• Submission of monthly reports by CHAs through the CBIS system.	• Use of data from reports and community feedback to be increased for problem solving and coaching	

*Review of implementation status adapted from the interagency iCCM benchmarks matrix (phase 1 = advocacy and planning; phase 2 = Pilot and Early Implementation; phase 3 = Expansion/Scale-up). The Community health program in Liberia has been extended from iCCM to a broad package of core plus additional services as defined in the revised community health policy.

## DISCUSSION

We learned important lessons from the process of the community health policy review, revision and program design which can inform future health systems development processes. The importance of five key processes emerged: 1) ensuring government leadership and ownership; 2) establishing a coordination mechanism and leveraging partnership support; 3) conducting evidence-informed planning to inform policy-makers; 4) using a systems approach through a participatory process to better inform policy shifts; and 5) strengthening community engagement and participation.

### Ensuring government leadership and ownership

Seeking ownership from the MOH and garnering support from senior management has been essential in driving the agenda for the redesign of the Community Health Program. Government leadership has been instrumental in driving the process, through strong commitment from the Minister of Health, endorsement from the Health Sector Coordination Committee, and political support from the cabinet. This level of ownership has ensured that all ministry divisions and departments have rallied around the process with the critical personnel taking lead of the various work streams for a harmonized program. Engaging local governments, community leaders, and program managers at the subnational level (county and districts) at an early stage of the policy and program development process has also been crucial [27,28].

### Establishing a coordination mechanism and leveraging partnerships

A strong national coordination structure involving all relevant technical divisions at the MOH, donors and developmental partners, and international and national NGOs is crucial to the design of a robust community health system. Leveraging the partnerships of all parties with a vested interest in community health programming ensures there is no fragmentation in service delivery, remuneration package for community cadres, and functional operational support systems such as supervision, monitoring, and supply chain for a comprehensive program [29,30]. Furthermore, the partnership enables sharing of available resources such that no one single entity is bearing the burden of such a resource intensive undertaking [30]. Sustained stakeholder engagement was achieved through regular meetings and information sharing.

### Conducting evidence-informed planning to inform policy makers

The bottleneck analysis framework provided an opportunity to engage stakeholders from different backgrounds to identify and prioritize context-specific health-systems barriers to the scale-up of community-based RMNCH interventions. It generated a renewed national-level focus on evidence-based decision-making. The bottleneck analysis exercise helped understand primary and secondary causes, inequities as well as identify and respond to the health system supply and demand-side bottlenecks that arise at the local level. This approach has been used by several countries for program planning and monitoring in order to accelerate progress toward the achievement of the SDGs [28,31,32].

### Systems approach for community health program design and implementation

Using the inter-agency benchmark, the community health program in Liberia was found to be in early implementation phase. Applying a health systems approach offers insights on how to develop a well-structured community-based program. This approach uses a framework that justifies program design and implementation from an evidence-based perspective [26,30], increases common understanding among stakeholders, helps prioritize investments across critical health system layers, and guides integration of community-based systems with national health systems. The health system approach facilitates the set-up of an integrated and potentially sustainable community-based program [30,33-35]. In Liberia, the analysis was combined with a qualitative assessment which helped to inform both the content of the program and the development of policy, as qualitative research is particularly useful in understanding why, how and under what conditions the policies, programs and projects work or fail to work [36].

### Strengthening community engagement and participation

Community engagement may have played a role in the decline in EVD transmission rates in Liberia [37,38]. It is recognized that community engagement is key to strengthening interventions that improve health outcomes [4,6]. Building a strong health system depends upon a greater role for communities in the delivery of services, mobilisation of demand, and increasing access to those most in need [39]. The health system in Liberia intends to build on the EVD response experience to ensure citizen engagement and community capacity building to identify and take corrective actions that are needed to manage future health threats [37,38]. Community engagement is one of the main programmatic shifts of the revised community health policy, although more efforts are required for effective implementation. Key considerations include setting up and revamping community networks and enhancing advocacy for social accountability and community-based participatory monitoring [39].

Health system bottleneck analysis and planning approaches which require key stakeholder consultations, have been recommended and used to inform the revision of national policies, identify gaps in service delivery and uptake of services by the community, or improve maternal, child, and neonatal health system outcomes in several countries, including India, Rwanda, Nigeria, and Tanzania [23,40-43]. In Sierra Leone, a government-led mixed approach was also used post-EVD for the development of the National Health Strategic Plan [44] and the 2016 Community Health Workers Policy [45]. The health system review process may have contributed to a better prepared community health system ready to manage shocks, although this warrants further investigation. In the context of the COVID-19 pandemic, a harmonized community health system contributing to surveillance, risk communication, community engagement, and service delivery has renewed importance.

### Strengths and limitations of the study

The bottleneck analysis and qualitative surveys provided rich data and elicited solutions to scale up community health in Liberia. Participants were given an equal voice to contribute ideas, which minimized the domination of the process by more confident or outspoken individuals. This research also discovered where perspectives converged, and what emerged as the real issues impacting the community health program. Although the study was not able to include all relevant actors (private sector, stakeholders from other line ministries), robustness was achieved by compiling, documenting, and including all data during the participatory analysis and validation meetings attended by most stakeholders. Despite these limitations, the in-depth description of the policy development and implementation process in this study provides a valuable contribution to the knowledge base on policy reforms for community-based health program in fragile and low resource settings.

## CONCLUSIONS

Stronger health systems are needed to accelerate the pace of ending preventable maternal, neonatal, and child deaths to contribute to the achievement of the SDGs [46]. Community health system strengthening requires that communities be viewed as active participants in the health system [47]. Since the EVD outbreak, concerted efforts have been made by African governments and partners to strengthen community health programs [47-50]. In settings with weak health systems, like Liberia, applying the government-led health system approach could better inform national Community Health Policy reviews. There is need for locally defined and context-specific strategies to improve access to quality primary health care, help communities prepare for future shocks, and minimize the impact of outbreaks like COVID-19.
